# CD4 Depletion in SIV-Infected Macaques Results in Macrophage and Microglia Infection with Rapid Turnover of Infected Cells

**DOI:** 10.1371/journal.ppat.1004467

**Published:** 2014-10-30

**Authors:** Luca Micci, Xavier Alvarez, Robin I. Iriele, Alexandra M. Ortiz, Emily S. Ryan, Colleen S. McGary, Claire Deleage, Brigitte B. McAtee, Tianyu He, Cristian Apetrei, Kirk Easley, Savita Pahwa, Ronald G. Collman, Cynthia A. Derdeyn, Miles P. Davenport, Jacob D. Estes, Guido Silvestri, Andrew A. Lackner, Mirko Paiardini

**Affiliations:** 1 Division of Microbiology & Immunology, Yerkes National Primate Research Center, Emory University School of Medicine, Atlanta, Georgia, United States of America; 2 Tulane National Primate Research Center, Tulane University School of Medicine, Covington, Louisiana, United States of America; 3 AIDS Cancer Virus Program, Frederick National Laboratory for Cancer Research, Leidos Biomedical Research, Inc., Frederick, Maryland, Maryland, United States of America; 4 Center for Vaccine Research, University of Pittsburgh, Pittsburgh, Pennsylvania, United States of America; 5 Department of Biostatistics & Bioinformatics, Rollins School of Public Health, Atlanta, Georgia, United States of America; 6 University of Miami Miller School of Medicine, Miami, Florida, United States of America; 7 Department of Medicine, University of Pennsylvania Perelman School of Medicine, Philadelphia, Pennsylvania, United States of America; 8 Emory Vaccine Center, Emory University School of Medicine, Atlanta, Georgia, United States of America; 9 Department of Pathology and Laboratory Medicine, Emory University School of Medicine, Atlanta, Georgia, United States of America; 10 Centre for Vascular Research, University of New South Wales, Kensington, New South Wales, Australia; Vaccine Research Center, United States of America

## Abstract

In rhesus macaques (RMs), experimental depletion of CD4^+^ T-cells prior to SIV infection results in higher viremia and emergence of CD4-independent SIV-envelopes. In this study we used the rhesus recombinant anti-CD4 antibody CD4R1 to deplete RM CD4^+^ T-cells prior to SIVmac_251_ infection and investigate the sources of the increased viral burden and the lifespan of productively infected cells. CD4-depleted animals showed (i) set-point viral load two-logs higher than controls; (ii) macrophages constituting 80% of all SIV vRNA^+^ cells in lymph node and mucosal tissues; (iii) substantial expansion of pro-inflammatory monocytes; (iv) aberrant activation and infection of microglial cells; and (v) lifespan of productively infected cells significantly longer in comparison to controls, but markedly shorter than previously estimated for macrophages. The net effect of CD4^+^ T-cell depletion is an inability to control SIV replication and a shift in the tropism of infected cells to macrophages, microglia, and, potentially, other CD4-low cells which all appear to have a shortened *in vivo* lifespan. We believe these findings have important implications for HIV eradication studies.

## Author Summary

CD4^+^ T-cells are both mediators of antiviral immune response and critical targets for HIV replication. We have previously shown that experimental depletion of CD4^+^ T-cells prior to SIV infection in rhesus macaques results in higher viremia and the emergence of CD4-independent SIV-envelopes. The findings reported in this new study of CD4 depletion address key unanswered questions about the phenotype, location, and lifespan of the sources of the increased viral replication in the absence of CD4^+^ T-cells. Altogether, our new data indicate that depletion of CD4^+^ T-cells prior to SIV infection results in activation of monocyte and massive infection of tissue-resident macrophages, which appear to be the predominant population of productively infected cells. Furthermore, our analysis of the slope of viremia decline after initiation of antiretroviral therapy suggests that the lifespan of these virus targets is markedly shorter than those previously estimated for macrophages. In summary, in the context of CD4^+^ T-cell depletion macrophages can be highly infectable, exhibit rapid turnover, and short *in vivo* lifespan. These finding raises a suggestive hypothesis that eradication of HIV from this reservoir could be enhanced by therapeutics able to modulate monocyte/macrophage turnover.

## Introduction

The interaction between HIV and CD4^+^ T-cells is complex, and may result in contrasting effects with respect to virus replication. On the one hand, CD4^+^ T-cells have a beneficial role as mediators of antiviral immune responses, both directly and by providing help for HIV-specific CD8^+^ T-cells and B cells [1]–[4]. On the other hand, CD4^+^ T-cells are key targets for infection and sustain virus replication [5], [6]. To better understand the relationship between CD4^+^ T-cell availability and HIV replication, we recently conducted a CD4^+^ T-cell depletion study in rhesus macaques (RMs) prior to SIV infection [7]. This previous study showed that antibody-mediated depletion of CD4^+^ T-cells was associated with increased virus replication and rapid disease progression [7]. Furthermore, using in vitro systems we demonstrated the emergence of CD4-independent SIV envelopes capable of mediating entry into cells expressing CCR5 without CD4. The absence of antibodies targeting conserved CD4-inducible epitopes has been proposed as one of the mechanisms allowing CD4-independent SIV to emerge in CD4-depleted RMs [8]. Of note, in that study one RM with the least effective CD4^+^ T-cell depletion showed the lowest viremia and survived throughout the entire study, suggesting that intermediate levels of CD4^+^ T-cells may be the ideal balance between the beneficial and harmful contribution of CD4^+^ T-cells to disease progression. This previous study raised some critical questions, including: (i) is partial depletion of CD4^+^ T-cells beneficial? (ii) What cells are the main sources of virus replication in the absence of CD4^+^ T-cells, and where are they located? (iii) What is the *in vivo* lifespan of these productively infected cells? And finally, (iv) can we identify correlates of the high viremia associated with CD4 depletion?

To answer these questions, we designed a new study where we used a concentration of the CD4 depleting antibody CD4R1 which generated variable levels of CD4^+^ T-cell depletion allowing us to test how CD4^+^ T-cell availability impacts SIV infection. Furthermore, we performed an extensive combined immunohistochemistry (IHC) and in situ hybridization (ISH) analysis on colon, jejunal and brain tissues collected at necropsy from the eight SIV-infected RMs - four CD4-depleted and four controls - included in *Ortiz A.M et al* [7]. In contrast to our current study, these animals were not ART-treated when euthanized. As such, they represent the ideal samples to investigate the presence and the phenotype of productively infected cells. Of note, both the present and the former studies were performed at the same facility, using the same animal species, virus, route and dose of infection.

We found that depletion of RM CD4^+^ T-cells prior to SIV infection is associated with dramatic changes in the course of disease, including post-peak viral load two-logs higher than undepleted controls, expansion of pro-inflammatory monocytes, and massive activation and infection of macrophages and microglia that appear to be the predominant population of productively infected cells. Finally, our analysis of the slope of viremia decline after initiation of antiretroviral therapy (ART) suggests that, in the absence of CD4^+^ T-cells and in the presence of high levels of activation, the lifespan of these virus targets is significantly longer than controls, but markedly shorter in comparison to those previously estimated for macrophages.

## Results

### Experimental design and extent of CD4^+^ T-cell depletion induced by CD4R1

**Figure 1:**
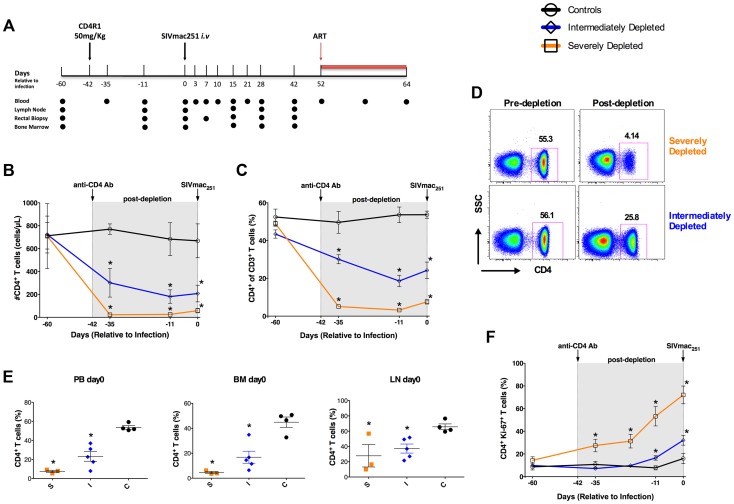
Study design and extent of CD4^+^ T-cell depletion induced by CD4R1. (**a**) Time line of the study indicating the days in which CD4R1 was administered, SIVmac251 infection performed, and ART initiated, as well as the experimental points at which blood (PB), bone marrow (BM), lymph nodes (LN) and rectal biopsies were collected. (**b,c**) Longitudinal levels (mean±S.E) of the circulating CD4^+^ T-cells expressed as absolute number (**b**) or fraction (**c**) of CD3^+^ T-cells in severely depleted (orange square; n = 3), intermediately depleted (blue diamond; n = 5) or undepleted control (black circle; n = 4) RMs. In both severely- and intermediately-depleted RMs, the percentage and absolute count of CD4^+^ T-cells were significantly lower (P<0.01) than controls at all experimental points post-depletion. (**d**) Representative flow plots showing the fraction of CD4^+^ T-cells pre- and post-depletion (day −11) in one severely (top) and one intermediately (bottom) depleted RMs. (**e**) Levels of CD4^+^ T-cells expressed as fraction of CD3^+^ T-cells in PB, BM and LN immediately before SIV infection (day 0) in severely depleted (S; n = 3), intermediately depleted (I; n = 5) or control (C; n = 4) RMs. In both severely- and intermediately-depleted RMs, the percentages of CD4^+^ T-cells were significantly lower (P<0.01) than controls. (**f**) Longitudinal levels (mean±S.E) of the fraction of circulating CD4^+^ T-cells expressed Ki-67 in severely depleted (n = 3), intermediately depleted (n = 5) or control RMs (n = 4). The gray box in the graphs of panels b, c, f indicates the post-depletion, pre-infection window. *P<0.01.

CD4^+^ T-cells were depleted in eight RMs using a single administration of CD4R1 antibody at 50 mg/kg, as recommended by the “NIH Nonhuman Primate Reagent Resource” protocol (**Figure 1a**). Of note, this regimen generated variable levels of CD4^+^ T-cell depletion in our pilot studies. Four untreated animals were included as controls. All 12 RMs were infected with SIVmac_251_ (i.v. 3,000 TCID_50_) six weeks post CD4R1 treatment, in order to avoid the possible confounding effect of direct antiviral activity of the CD4R1 antibody. At day-52 post-infection (p.i.) all animals were treated with a three-drugs antiretroviral regimen (PMPA, FTC, raltegravir). Blood, bone marrow aspirate (BM), lymph nodes (LN) and rectal biopsies (RB) were collected longitudinally and at necropsy (**Figure 1a**).

The efficacy of CD4^+^ T-cell depletion was determined in blood and tissues by flow cytometry. Figure 1 shows the longitudinal levels (mean±S.E) of the circulating CD4^+^ T-cells expressed as the absolute number (**b**) or fraction of CD3^+^ T-cells (**c**). Treatment with 50 mg/kg CD4R1 resulted in a severe depletion in three RMs (orange), but an intermediate depletion in the remaining five treated animals (blue). Of note, there was no association between the extent of CD4^+^ T-cell depletion and the age, sex, and weight of the animals. Representative flow plots of the percentage of CD4^+^ T-cells pre- and post-depletion are shown in **Figure 1d** for a severely (top) and an intermediately (bottom) depleted RM. Severe depletion was characterized by a nadir CD4^+^ T-cell frequency of 3.18±0.356% (93.5% depletion from baseline), and CD4^+^ T-cell counts of 25.64±6.877 cells/µL (**Figure 1b,c**). Intermediately-depleted animals had a nadir CD4^+^ T-cell frequency of 18.6±3.021% (57% depletion from baseline) and CD4^+^ T-cell counts of 181.2±58.98 cells/µL. In both severely- and intermediately-depleted RMs, the percentage and absolute count of CD4^+^ T-cells were significantly lower (P<0.01) than controls at all experimental points post-depletion (D-35, D-11, D0; **Figure 1b,c**). The same gradient of CD4^+^ T-cell depletion was confirmed in BM and LN tissues (**Figure 1e**). Since we used an anti-CD4 antibody clone for immunophenotyping that is not cross-blocked by CD4R1, and additionally found a significant reduction in the fraction of CD3^+^ T-cells and no selective increase in the fraction of CD3^+^CD4^−^CD8^−^ T-cells, we are confident that the loss of CD4^+^ T-cells observed following CD4R1 administration is indeed a true depletion of these cells, rather than a masking. As expected based on previous studies [7], depletion of CD4^+^ T-cells was minimal at mucosal sites. In severely-depleted RMs, depletion of CD4^+^ T-cells induced high levels of proliferation, with in average 72% of the remaining CD4^+^ T-cells expressing Ki-67 at D0 (**Figure 1f**). Although we cannot exclude the contribution of other mechanisms, including reactivation of latent infections, we interpreted this high proliferation as an attempt of the immune system to reconstitute the depleted CD4^+^ T-cell compartment.

In summary, the variable efficacy of CD4^+^ T-cell depletion achieved in this study allowed us to study SIV viral dynamics and disease progression in RMs with normal, low or extremely low levels of circulating and tissue resident CD4^+^ T-cells, therefore representing an ideal model to address how CD4^+^ T-cells impact SIV infection.

### Lack of post-peak viral decline in CD4-depleted SIV-infected RMs

**Figure 2:**
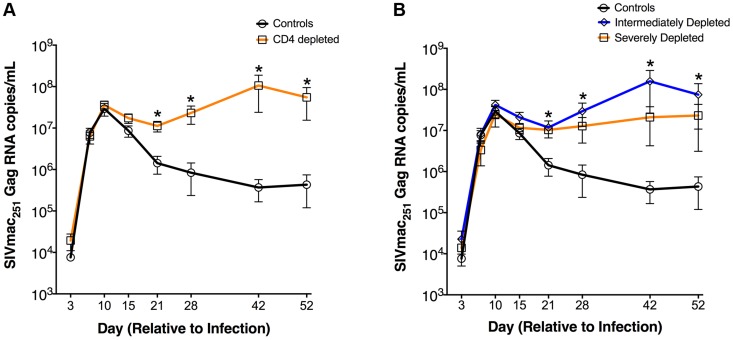
Increased plasma viremia in CD4-depleted SIV-infected RMs. Longitudinal levels (mean±S.E) of viral load, expressed as number of SIVmac251Gag RNA copies per mL of plasma, in CD4-depleted (orange square; n = 8) vs. undepleted control (black circle; n = 4) RMs (**a**) as well as in severely depleted (orange square; n = 3), intermediately depleted (blue diamond; n = 5) or undepleted control (black circle; n = 4) RMs (**b**). In both severely- and intermediately-depleted RMs, viral loads were significantly higher than controls from day 21 to day 52 post-infection. *P<0.01.

Six weeks post CD4R1 administration all twelve RMs were infected with SIVmac_251_ (i.v. 3,000 TCID_50_). Peak viral load (day 10 p.i.) was similar between CD4-depleted and control RMs (**Figure 2a,b**). However, whereas control animals showed a rapid drop in viral load after the peak, CD4-depleted animals maintained viremia close to peak levels and ∼2-logs higher than controls in later infection. This difference in viral load was statistically significant (P<0.001) at each experimental point between day 21 and day 52 p.i. (**Figure 2a,b**). Intermediately-depleted animals exhibited viral kinetics remarkably similar to those of severely-depleted animals (**Figure 2b**). Furthermore, following SIV infection the absolute numbers of CD4^+^ (a), CD4^+^Ki-67^+^ (b) or CD4^+^CCR5^+^ (c) T-cells were comparable in intermediately-depleted RMs when compared to severely-depleted animals, and significantly lower than those found in controls at all the experimental points (**Figure S1**). Since severely- and intermediately-depleted RMs had comparable viremia and numbers of activated/proliferating T-cells, for the remaining of the study all eight treated animals were grouped together and defined as CD4-depleted RMs.

These results show that a partial depletion of CD4^+^ T-cells is sufficient for the establishment of an infection phenotype of persistently high viremia. Furthermore, the presence of a 2-log higher viremia together with a dramatic loss of CD4^+^ T-cells suggests a different source of viral burden in CD4-depleted animals.

### Expansion of activated monocytes in CD4-depleted SIV-infected RMs

**Figure 3:**
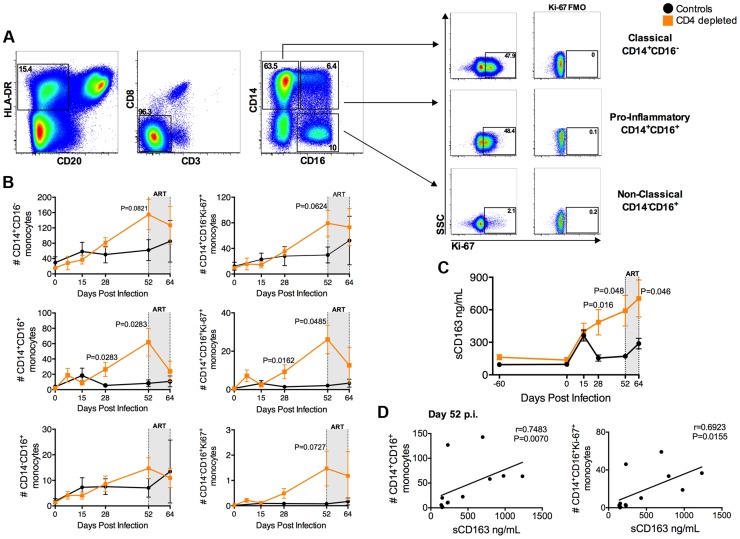
Expansion of activated, pro-inflammatory monocytes in CD4-depleted SIV-infected RMs. (**a**) Representative flow plots of different monocyte subsets as defined by CD14 and CD16 expression. Levels of Ki-67 and FMO controls are represented for classical (CD14^+^CD16^−^), pro-inflammatory (CD14^+^CD16^+^), and non-classical (CD14^−^CD16^+^) monocytes. (**b**) Quantification of total and Ki-67^+^ blood monocyte subsets (cells/µl) in SIV-infected CD4-depleted (orange square; n = 8) and undepleted control (closed circle; n = 4) RMs. In depleted RMs, the number of total and proliferating CD14^+^CD16^+^ monocytes was significantly higher than undepleted controls at day 28 and 52 post-infection. (**c**) In CD4-depleted RMs (orange square; n = 8) plasma levels of soluble CD163 (sCD163) were significantly higher than undepleted controls (closed circle; n = 4) at day 28 and day 52 post-infection, as well as after 12 days of ART. (**d**) Plasma level of sCD163 significantly correlates with the numbers of CD14^+^CD16^+^ and of CD14^+^CD16^+^Ki-67^+^ monocytes in all SIV-infected RMs (day 52 p.i.; n = 12; Spearman rank correlation tests). The gray box in the graphs of panels b and c indicates the 12 days of ART.

Increased activation and turnover of monocytes predict progression to AIDS in SIV-infected RMs even better than CD4^+^ T-cell number [9]–[11]. Hence, we quantified the levels and Ki-67 expression of the monocyte subsets in CD4-depleted and control RMs. Monocytes were defined as classical (CD14^+^CD16^−^), pro-inflammatory (CD14^+^CD16^+^), and non-classical (CD14^−^CD16^+^) based on the expression of CD14 and/or CD16 (**Figure 3a**). In the control animals, stable levels of all monocyte subsets at day 52 p.i. followed their initial expansion. However, classical and pro-inflammatory monocytes in CD4-depleted RMs continued to increase, with numbers of CD14^+^CD16^+^ monocytes significantly higher than those of controls at days 28 (P = 0.0283) and 52 (P = 0.0283) p.i. (**Figure 3b**). Furthermore, and consistent with an activated/pro-inflammatory status and an increased output from bone marrow, the number of CD14^+^CD16^+^ monocytes expressing Ki-67^+^ in CD4-depleted RMs was significantly higher as compared to controls at days 28 (P = 0.0162) and 52 (P = 0.0485) p.i. (**Figure 3b**). At day 52 p.i. CD4-depleted RMs also have a higher number of proliferating CD14^+^CD16^−^ and CD14^−^CD16^+^ monocytes than controls, although the difference was not statistically significant (**Figure 3b**). In CD4-depleted RMs plasma levels of soluble CD163 (sCD163), a marker of monocyte activation associated with rapid disease progression [9], [12], [13], were significantly higher than in controls at day 28 (p = 0.016) and day 52 (p = 0.048) p.i. (**Figure 3c**). Of note, at day 52 p.i., levels of sCD163 strongly correlated with the numbers of total (r = 0.7483, P = 0.0070) and Ki-67^+^ (r = 0.6923, P = 0.0155) CD14^+^CD16^+^ monocytes (**Figure 3d**), as well as with viremia (r = 0.8322; P = 0.0013) (**Figure S2**). Thus, depletion of CD4^+^ T-cells prior to SIV infection results in increased number, activation and turnover of monocytes during early SIV infection. As indicated in Figure 1a, all 12 SIV-infected RMs were started on ART at day 52 p.i. The numbers of pro-inflammatory monocytes (CD14^+^CD16^+^; P = 0.0145) and their levels of Ki-67 expression (P = 0.0189) after 12 days of ART (day 64 p.i) were significantly decreased as compared to pre-ART (day 52 p.i.) levels, and become comparable to those found in controls (**Figure 3b**). Classical (CD14^+^CD16^−^) and non-classical (CD14^−^CD16^+^) monocyte levels were not significantly affected by ART (**Figure 3b**). Levels of sCD163 remained significantly higher in CD4 depleted animals than controls after 12 days of ART (**Figure 3c**). Of note, the interpretation of the effects of ART in the aforementioned parameters, in particular for sCD163, is complicated by the fact that we were able to only treat the animals for a short period. Indeed, and perhaps as a consequence of the fact that CD4-depletion resulted in very high virus replication, seven of the eight depleted RMs had to be euthanized for AIDS-related reasons briefly after ART initiation (**Table S1**).

### Macrophages are highly infected and constitute the major contributors to systemic viremia in CD4-depleted RMs

**Figure 4:**
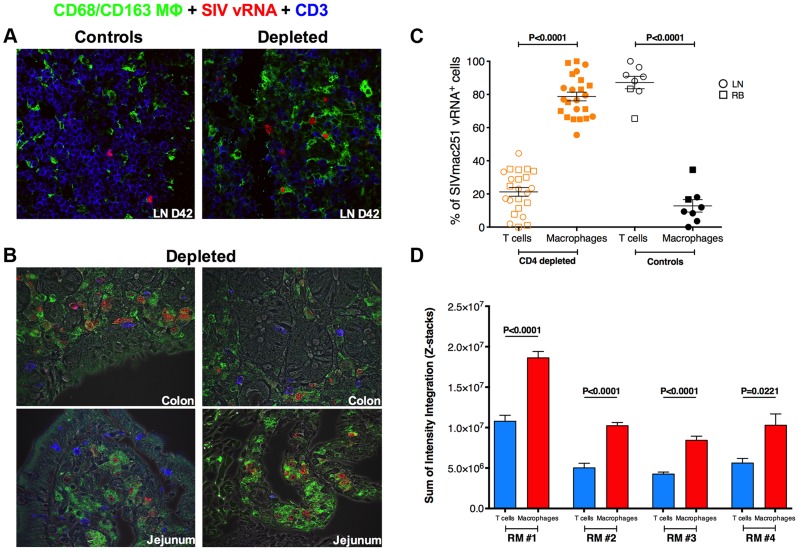
Massive SIV infection of LN and intestinal macrophages in CD4-depleted RMs. (**a**) Immunofluorescence staining for T cell (CD3; blue) and macrophage (CD68 and/or CD163; green) lineage markers combined with in situ hybridization for SIV vRNA (red) in the LN isolated at day 42 post-infection from one representative undepleted control (left) and one CD4-depleted RM. The vast majority of SIV vRNA^+^ cells express CD3 in undepleted control but express CD68/CD163 in CD4-depleted RM. (**b**) The same staining is shown for colon (top) and jejunum (bottom) tissues in two representative CD4-depleted animals. (**c**) Quantitative image analysis of LN and mucosal tissues showing the fraction of SIV vRNA^+^ cells that express CD3 or CD68/CD163 in CD4-depleted (n = 12; the 8 animals in this study plus the 4 in *Ortiz et al 2011*) or undepleted control RMs (n = 6; two SIVmac251 infected animals belonging to a different study were added as controls). (**d**) Relative abundance of SIV vRNA^+^ in productively infected T cells and macrophages, determined by measuring the volumetric sum of the SIV vRNA^+^ intensity integration values *in situ* from confocal images collected under identical laser settings, is shown in four CD4-depleted RMs. Macrophages on average have higher per cell SIV vRNA^+^ content compared to CD4^+^ T cells within the same host. Statistical analyses were determined by Mann-Whitney test.

We next investigated the levels of SIV infection of macrophages in peripheral lymph node and intestine from CD4-depleted and control RMs by immunofluorescence staining for cell lineage markers combined with fluorescence in situ hybridization (F-ISH) for SIV vRNA. Since monocyte/macrophage express CD4, we used CD3 to determine the infection frequency of CD4^+^ T-cells. At day 42 p.i. SIV vRNA^+^ cells were more frequent in the LN of CD4-depleted animals as compared to controls (**Figure 4a**), consistent with the ∼2-log higher plasma viremia found at the same experimental point. The vast majority of SIV vRNA^+^ cells expressed the T cell marker CD3 in undepleted controls, but macrophage markers (CD68 and/or CD163) in CD4-depleted RMs. We then performed the same F-ISH staining on day 42 rectal biopsies from the RMs included in the current study as well as on colon and jejunal tissues at necropsy from the four CD4-depleted animals included in *Ortiz A.M et al* [7]. In contrast to our current study, those latter animals were not ART-treated and thus showed high viremia when euthanized. The same phenomena of increased levels of total SIV vRNA^+^ cells and expression of macrophage markers by infected cells were observed in the intestine of CD4-depleted (**Figure 4b**), but not of control RMs. Quantitative image analysis of LN and intestinal tissues showed that in undepleted controls more than 80% of SIV vRNA^+^ cells were CD3^+^ T-cells, while in CD4-depleted RMs ∼80% of SIV vRNA^+^ cells were CD68^+^ and/or CD163^+^ macrophages (**Figure 4c**).

Although the majority of infected cells in CD4-depleted animals were macrophages, it is possible that infection of these cells was a result of high viral loads, and they did not contribute substantially to the total viral load because they produced little virus per infected cell. In order to determine whether viral production from macrophages was likely to contribute to the total viral load, we sought to compare the relative abundance of SIV vRNA in productively infected CD4^+^ T cells to that of macrophages in four CD4-depleted RMs. We measured the volumetric sum of the SIV vRNA intensity integration values in defined productively SIV infected CD3^+^ and CD68/CD163^+^ cells *in situ* from confocal images collected under identical laser settings. Using this approach we were able to demonstrate that, within a particular host, macrophages have similar or even higher (on average two-fold more) SIV vRNA content per cell than productively infected CD4^+^ T-cells (**Figure 4d**).

Altogether, these data indicate that depletion of CD4^+^ T-cells prior to SIV infection results in activation of monocyte and massive infection of tissue-resident macrophages. These infected macrophages have higher levels of SIV vRNA than infected CD4^+^ T cells, and, since they constitute 80% of all infected cells in CD4-depleted animals, they are most likely the major source of systemic viremia in these animals.

### Massive activation and productive infection of microglia in CD4-depleted RMs

**Figure 5:**
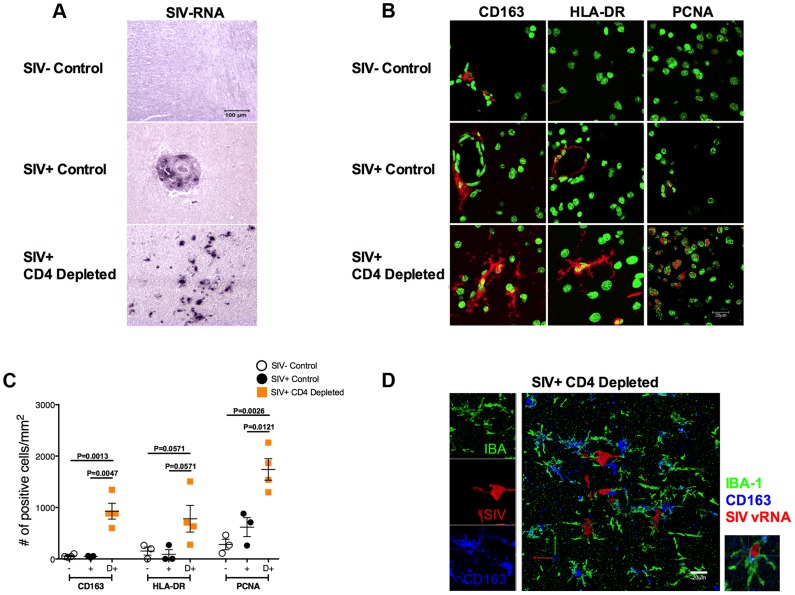
Massive activation and productive infection of microglia in CD4-depleted RMs. (**a**) ISH showing the levels of SIV vRNA^+^ cells in brain from a representative SIV-uninfected control (top), SIV-infected control (middle) and CD4-depleted SIV-infected (bottom) animal. The amount of SIV vRNA^+^ cells was markedly higher in CD4-depleted animals than in controls. (**b**) Immunofluorescence staining for CD163 (left panels), HLA-DR (middle panels) and proliferating cell nuclear antigen (PCNA; right panels) in the parenchyma of one representative SIV-uninfected control (top), one SIV-infected control (middle) and one SIV-infected CD4-depleted (bottom) RM. Nuclei are stained in green; markers of interest in red. (**c**) Quantitative analyses showing the number of cells per mm^2^ of tissue that stained positively for the markers of interest. Increased expression of CD163, HLA-DR and PCNA was found in CD4-depleted animals (orange square; n = 4) when compared to both groups of controls (SIV- open circle, n = 3; SIV+ closed circle, n = 3). (**d**) Single and combined staining for IBA-1 (green), CD163 (blue), and SIV-vRNA (red) in the brain of one representative SIV-infected CD4 depleted RM. The box on the right is a magnification of a microglial cell (IBA-1^+^) that expresses CD163 and is productively infected (SIV vRNA^+^).

We then investigated if the observed high viremia, monocyte activation, and infection of macrophages in peripheral tissues were associated with central nervous system (CNS) virus dissemination and pathology. First, we investigated the presence of infected cells in brain sections collected at necropsy from the CD4-depleted and non-depleted (SIV^+^ controls) SIV-infected RMs included in our previous study (in contrast to the current study, these animals were not treated with antiretroviral drugs at the time of necropsy [7]). For the SIV negative controls, we used brain sections from historically SIV-uninfected RMs present at the Tulane National Primate Research Center (SIV^−^ controls). In CD4-depleted RMs SIV vRNA^+^ cells were found throughout the parenchyma (**Figure 5a**) and had a stellate morphology typical of microglia. The high number of infected cells led us to evaluate, in the same brain sections, the levels of activation by IHC/IF staining for CD163, HLA-DR, and proliferating cell nuclear antigen (PCNA, a marker of perivascular macrophages that has been used as a marker of DNA repair in macrophages [14]). As shown in the representative images (**b**) and in the quantitative analysis (**c**) of **Figure 5**, the expression of CD163 (P = 0.0013; P = 0.0047), HLA-DR (P = 0.0571; P = 0.0571), and PCNA (P = 0.0026; P = 0.0121) in cells with location and stellate morphology typical of microglia were significantly higher in CD4-depleted than SIV-uninfected or SIV-infected controls. The surprising finding that a significant number of microglia are activated and productively infected was confirmed by triple fluorescent labeling for SIV vRNA (red), CD163 (blue) and the microglia-specific marker IBA-1 [15] (green) (**Figure 5d**). Quantification of these stainings showed numbers of SIV vRNA^+^ IBA-1^+^ microglia markedly higher in CD4-depleted compared to non-depleted SIV-infected animals (11.3±8.4 vs. 0.2±0.2; P = 0.0286). Of note, we found a direct correlation between the number (cells/mm^2^) of SIV vRNA^+^ cells and PCNA^+^ cells in the eight CD4-depleted animals (r = 0.8289, P = 0.0302).

Collectively, these results indicate that depletion of CD4^+^ T-cells in RMs results in massive activation and infection of microglia.

### Short lifespan of productively infected cells in CD4-depleted, SIV-infected RMs

One way of testing the longevity of infected cells is to block new rounds of infection with ART and measure the rate of viral decay (which reflects the death rate of productively infected cells present at the time of treatment). Thus, if infected macrophages have a longer survival than infected CD4 T cells, we should expect to see a much slower decay of virus under therapy in CD4-depleted RMs, where infected macrophages are the major source of systemic viremia.

**Figure 6:**
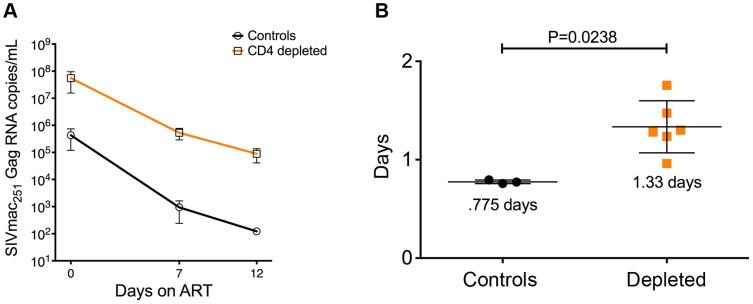
Viral decay kinetics in CD4-depleted and control RMs. (**a**) Longitudinal levels (mean±S.E) of viral load, expressed as number of SIVmac251Gag RNA copies per mL of plasma, in ART-treated CD4-depleted (orange square; n = 6) and undepleted control (closed circle; n = 3) RMs. Viral load was assessed at ART-initiation (day 0) as well as at day 7 and day 12 on-ART. (**b**) In vivo lifespan of the predominant population of productively infected cells was estimated for CD4-depleted and undepleted control RMs based on the viral load changes between day 0 and day 7 (although the same conclusions were reached if we included day 12) showed in (**a**). Two CD4-depleted RMs were sacrificed before day 7 on-ART (one at day 3 and one at day 6 on-ART; see Table S1); thus, the graph includes the six CD4-depleted animals for which data were available at day 7 on-ART.

To directly estimate the *in vivo* lifespan of the productively infected cells, all 12 SIV-infected RMs were started on ART at day 52 p.i. We then measured and modeled the slope of viral load decay during ART as described in previous studies [16]–[19]. As described above, seven of the eight depleted RMs had to be euthanized for AIDS-related reasons briefly after ART initiation (**Table S1**). As a result of this, we were able to perform only three sample collections, one at ART initiation (day 0) and the other two at day 7 and day 12 on-ART (**Figure 6a**). Since at day 12 on-ART we already lost three CD4-depleted animals, the lifespan of productively infected cells was estimated based on viral load changes between day 0 and day 7, although the same conclusions were reached if we used the smaller subset of animals alive at day 12. As expected, the viral decay in control animals was rapid, with an estimated half-life of infected cells of 0.775±0.01 days, which was consistent with previous estimates. The lifespan of productively infected cells was significantly longer in the CD4-depleted animals (1.33±0.108 days, P = 0.0238; **Figure 6b**), but was considerably shorter than expected for HIV infected macrophages [20]–[22]. Thus, in CD4-depleted RMs, macrophages constitute the main productively infected cell type, produce the majority of virus, and show a relatively short *in vivo* lifespan. Further analysis of cell death in a subset of animals showed a statistically significant higher number of TUNEL positive cells (the vast majority of which express CD163) in the brain parenchyma in CD4-depleted RMs than in controls (p = 0.0439) and CD4-depleted animals on-ART (p = 0.0002) (**Figure S3a**). The increased cell death was not Caspase-3 mediated since the number of cells positive for the active form of Caspase-3 was comparable between SIV-infected CD4-depleted RMs and controls (Figure S3b).

## Discussion

We previously showed that a severe depletion of CD4^+^ T-cells is associated with increased viremia, emergence of CD4-independent viruses and rapid progression to AIDS in SIV-infected RMs [7]. In this new study we used CD4R1, a rhesus recombinant anti-CD4 depleting antibody, to achieve a gradient of CD4^+^ T-cells *in vivo*. This strategy allowed us to experimentally infect with SIV_mac251_ RMs with normal, low or very low level of CD4^+^ T-cells in blood and lymphoid tissues. The critical questions we aimed to answer in this study were the following: (i) is partial depletion of CD4^+^ T-cells beneficial? (ii) What cells are the main sources of virus replication in the absence of CD4^+^ T-cells, and where are they located? (iii) What is the *in vivo* lifespan of these productively infected cells? And (iv) can we identify correlates of the high viremia associated with CD4 depletion? Answering these questions may have important implications for our understanding of HIV pathogenesis and latency.

Viral loads were indistinguishable between CD4-depleted and control RMs for the first 14 days p.i. However, severely- and intermediately-depleted RMs maintained viremia ∼2-logs higher than undepleted controls at all subsequent experimental points. Thus, partial depletion of CD4^+^ T-cells prior to infection did not result in a more benign course of infection and was sufficient to generate the higher viremia phenotype. CD4R1 was efficient in depleting CD4^+^ T-cells from blood, BM, and LN but not from mucosal sites. Thus, mucosal CD4^+^ T-cells may be responsible for maintaining peak viral loads in depleted RMs similar to that in controls, before being severely depleted in the first weeks of infection due to the high levels of SIV replication. Of note, our data on the level of proliferating cells does not support the possibility that CD4-depleted RMs have higher numbers of T-cells that can support viral replication as a result of the high level of activation/proliferation in the remaining CD4^+^ T-cells. Thus, in RMs the association of viremia that is ∼2-logs higher with a profound loss of CD4^+^ T-cells is consistent with an alternative critical source of virus replication.

The observed high viremia in the context of very low levels of CD4^+^ T-cells prompted us to investigate the susceptibility of macrophages/myeloid cells to SIV infection. Remarkably, we showed directly *in situ* that CD4-depletion induces a significant shift in the nature of productively infected cells, with 80% of SIV vRNA^+^ cells in LN and mucosal tissues being CD3^+^ T-cells in undepleted controls but CD68/CD163^+^ macrophages in CD4-depleted RMs. It has recently been described that increased activation and rapid turnover of monocytes, in particular those with a CD14^+^CD16^+^ phenotype, associates with macrophage destruction in tissues and predicts the tempo of progression to AIDS in SIV-infected RMs [9]–[11]. In response to activation, CD163 is cleaved from the cellular surface of monocytes/macrophages and is shed as sCD163 [23]. Recently, sCD163 has been indicated as a strong correlate of monocyte activation and turnover as well as disease progression in SIV-infected RMs [9]. Consistent with these studies, CD4-depleted RMs, but not undepleted controls, experienced a substantial increase in the number of circulating CD14^+^CD16^+^ monocytes. Furthermore, plasma levels of sCD163 were significantly higher in CD4-depleted than in undepleted control RMs, and directly correlated with viral load as well as with the numbers of total and proliferating CD14^+^CD16^+^ monocytes. Although *in vivo* BrdU labeling studies were not performed, the findings of higher frequency of Ki-67^+^ monocytes and sCD163 plasma levels are consistent with a model in which CD4-depletion prior to SIV infection results in increased activation/turnover of monocytes.

The key findings we described in CD4-depleted animals - high viral load, expansion of pro-inflammatory monocyte, increased level of sCD163, and massive infection of tissue-resident macrophages/myeloid cells – are all potential markers of CNS infection [7], [9], [13], [24]–[27]. In this study, CD4-depleted animals were euthanized during ART, thus the analyzed brain tissues did not represent the ideal samples to investigate the presence of productively infected cells. To overcome this limit, we stained brain sections collected in our previous study of CD4^+^ T-cell depletion that did not include ART [7]. It is worth mentioning that both the present and the former studies were performed at the same facility, using the same species, virus, route and dose of infection. Furthermore, the high viremia and rapid disease progression phenotype was remarkably similar between the two studies. Our combined IHC/ISH approach provided three surprising results. First, a large fraction of microglial cells from CD4-depleted animals expressed high levels of activation/proliferation/DNA repair markers, such as CD163, HLA-DR and PCNA, that were absent or exclusively present in perivascular macrophages in brain tissue of uninfected RMs and undepleted SIV-infected RMs. Second, the amount of SIV vRNA^+^ cells was remarkably higher in the brain of CD4-depleted RMs when compared to undepleted SIV-infected controls, thus consistent with what we found in LN and RB tissues. Third, and uniquely for the CD4-depleted RMs, SIV vRNA^+^ cells include not only perivascular macrophages but also cells with anatomic location, morphology, and phenotype typical of microglia. Demonstrable *in vivo* infection of microglia in SIV-infected RMs, particularly at the extent found here, is an exceedingly rare event even in the well-developed models of accelerate SIVE induced by depletion of CD8^+^ T-cells or by joint infection with an immune-suppressive and a neurovirulent SIV-variants [9], [15], [28].

Based on the paucity of CD4^+^ T-cells and the infection of macrophages/myeloid cells, which are typically thought to be long-lived cells, we hypothesized that in CD4-depleted RMs viral load decay during ART would be slower when compared to undepleted controls. In CD4-depleted RMs the lifespan of the main productively infected cells was 1.3 days, considerably shorter than what is generally expected for macrophages [20], [21], [29].

Two caveats must be considered when discussing this result. The first is that the decay model assumes that viral load is at steady-state and ART is equally effective at inhibiting viral replication in depleted and control animals. The second is related to a specific limitation of our study. Due to the poor clinical conditions of CD4-depleted RMs at ART initiation and their rapid disease progression, we could not sample these animals as frequently and as long as originally planned. Thus, the estimates of viral decay rates are based on a few relatively early experimental time points on-ART. However, given the rapid rate of decay in both groups, we were able to measure a substantial drop in viral load over seven days in both cases, and observed a significant difference in the decay rate of infected cells between control and CD4-depleted animals. These caveats notwithstanding, we concluded that the bulk of the available data are consistent with the idea that– in the context of CD4^+^ T-cell depletion and high levels of activation/inflammation—macrophages can be highly infectable, exhibit rapid turnover, and short *in vivo* lifespan when infected. In this regard, our data are consistent with recent findings indicating that rapid turnover of monocyte/macrophages determined by *in vivo* Brdu labeling predicted AIDS progression better than viral load or lymphocyte activation [9], [10], [30]. An alternative interpretation of the short lifespan of productively infected cells in CD4-depleted RMs is that the few remaining productively infected CD4^+^ T-cells produce much more virus than productively infected macrophages on a per cell basis. Thus in this scenario, T-cells could still constitute the major source of virus production measured in the plasma. However, this seems extremely unlikely because the differences in virus production would need to be dramatically skewed in favor of productively infected CD4^+^ T-cells, considering that they represent only 20% of all productively infected cells. In fact, when we measured *in situ* the relative volumetric abundance of SIV vRNA within CD3^+^ T cells and CD68/CD163^+^ macrophages, we found that macrophages on average have even higher per cell SIV vRNA content (∼2-fold greater) compared to productively infected CD4^+^ T-cells within the same host. With macrophages representing approximately 80% of all productively infected cells in all examined tissues, and with higher levels of SIV vRNA per cell in infected macrophages, it is safe to conclude that macrophages are the main productively infected cells in CD4-depleted animals. It is important to note that, due to the short length of ART-treatment, our data do not exclude the existence of a subpopulation of SIV-infected, long-lived macrophages. However, this would constitute a very small percentage of total viral production at most, because of the observed rapid viral load seen in CD4-depleted animals.

In conclusion, our study demonstrates that, in SIV-infected RMs, the net effect of CD4^+^ T-cell depletion is the inability to control SIV replication and a shift in the pattern of infected cells from CD4^+^ T-cells to macrophages, microglia, and, potentially, other CD4-low cells. These findings have important implications for functional cure and eradication studies as they indicate that macrophages and microglia can be critical target for virus infection in the context of a severely compromised immune system. Furthermore, our finding that HIV-infected macrophages can be short-lived if highly activated raises a suggestive hypothesis that eradication of HIV from this reservoir could be enhanced by therapeutics able to modulate monocyte/macrophage turnover.

## Materials and Methods

### Ethics statement

All animal experimentations were conducted following guidelines established by the Animal Welfare Act and the NIH for housing and care of laboratory animals and performed in accordance with Institutional regulations after review and approval by the Institutional Animal Care and Usage Committees at the Yerkes National Primate Research Center (YNPRC). All efforts were made to minimize suffering. All the blood and tissue collections were obtained from RMs housed at the Yerkes National Primate Research Center, which is accredited by American Association of Accreditation of Laboratory Animal Care. RMs are fed standard monkey chow (Jumbo Monkey Diet 5037, Purina Mills, St Louis, MO) twice daily. Consumption is monitored and adjustments are made as necessary depending on sex, age, and weight so that animals get enough food with minimum waste. SIV-infected RMs are singly caged but have visual, auditory, and olfactory contact with at least one social partner, permitting the expression of non-contact social behavior. The YNPRC enrichment plan employs several general categories of enrichment. Animals have access to more than one category of enrichment. IACUC proposals include a written scientific justification for any exclusions from some or all parts of the plan. Research-related exemptions are reviewed no less than annually. Clinically justified exemptions are reviewed more frequently by the attending veterinarian. This study was performed in strict accordance with the recommendations in the Guide for the Care and Use of Laboratory Animals of the National Institutes of Health, a national set of guidelines in the U.S. and also to international recommendations detailed in the Weatherall Report (2006). This work received prior approval by the Institutional Animal Care and Use Committees (IACUC) of Emory University (IACUC protocol #2000353, entitled “Homeostasis of CD4+ T cells in non-human primates”). Appropriate procedures were performed to ensure that potential distress, pain, discomfort and/or injury was limited to that unavoidable in the conduct of the research plan. The sedative Ketamine (10 mg/kg) and/or Telazol (4 mg/kg) were applied as necessary for blood and tissue collections and analgesics were used when determined appropriate by veterinary medical staff.

### Animals, CD4 depletion and antiretroviral therapy

Twelve female RMs were included in this study. Eight of them were treated with a single administration of the anti-CD4 antibody CD4R1 at 50 mg/kg (intravenous), as recommended by the “NIH Nonhuman Primate Reagent Resource” protocol. CD4R1 has rhesus constant regions and rhesus variable framework sequences. Only the CDRs (and a few amino acids critical for Ig conformation) are derived from the original mouse antibody. Four untreated animals were included as controls. All 12 RMs were infected with SIVmac_251_ (i.v. 3,000 TCID_50_) six weeks post CD4R1 treatment. At D52 post-infection all animals were treated once a day with Tenofovir (30 mg/kg; s.c.), Emtricitabine (30 mg/kg; s.c.) and Raltegravir (100 mg; oral). Furthermore, we used paraffin embedded tissues, including brain, collected at necropsy from the eight SIV-infected RMs (four CD4-depleted and four controls) included in *Ortiz A.M et al* [7]. All animal experimentations were conducted following guidelines established by the Animal Welfare Act and the NIH for housing and care of laboratory animals and performed in accordance with Institutional regulations after review and approval by the Institutional Animal Care and Usage Committees at the Yerkes National Primate Research Center (YNPRC). All efforts were made to minimize suffering.

### Sample collection and processing

Collections and processing of blood, bone marrow aspirate (BM), lymph nodes (LN) and rectal (RB) biopsies were performed longitudinally and at necropsy as previously described [31]–[34]. Briefly, blood samples have been used for a complete blood count, and plasma separated by centrifugation within 1 h of phlebotomy. Peripheral blood mononuclear cells were prepared by density gradient centrifugation. BM aspirates were performed using an aspiration kit to remove approximately 1 mL. For rectal biopsies, an anoscope has been placed a short distance into the rectum and up to 20 pinch biopsies obtained with biopsy forceps. RB-derived lymphocytes have been isolated by digestion with 1 mg/ml collagenase for 2 h at 37°C, and then passed through a 70-µm cell strainer to remove residual tissue fragments. For lymph node biopsies, the skin over the axillary or inguinal region have been clipped and surgically prepared. An incision has been made over the LN, which has been exposed by blunt dissection and excised over clamps. Biopsies have been homogenized and passed through a 70-µm cell strainer to mechanically isolate lymphocytes. All samples were processed, fixed (1% paraformaldehyde), and analyzed within 24 hours of collection.

### Flow cytometric analysis

Twelve-parameter flow cytometric analysis was performed on WB, LN, RB and BM derived cells according to standard procedures using a panel of monoclonal antibodies that we and others have shown to be cross-reactive with RM [35]–[37]. Predetermined optimal concentrations were used of the following antibodies: anti-CD3-Alexa700 (clone SP34-2), anti-CD3-APC-Cy7 (clone SP34-2), anti-CD4-Pacific Blue (clone OKT4), anti-CD8-APC-Cy7 (clone SK1), anti-CD95-PE-Cy5 (clone DX2), anti-CCR5-APC (clone 3A9), anti-Ki-67-Alexa700 (clone B56), anti-Ki-67-FITC (clone B56), anti-CD14-Pe-Cy7 (clone M5E2), anti-CD16-BV421 (clone 3G8), anti-CD62L-PE (clone SK11), anti-CCR7-PE-Cy7 (Clone 3D12) (all from BD Pharmingen); anti-CD28-ECD (clone CD28.2) (Beckman Coulter); anti-CD8-Qdot705 (clone 3B5) and Aqua Live/Dead amine dye-AmCyan (Invitrogen). Intracellular staining for Ki-67 was performed at room temperature for 30 minutes following permeabilization with cytofix/cytoperm (BD Bioscience). Flow cytometric acquisition was performed on an LSRII cytometer driven by the FACS DiVa software. Analysis of the acquired data was performed using FlowJo software (TreeStar) and graphs were prepared using Prism version 6.0 (GraphPad).

### Determination of viral load RNA

Quantitative real-time RT-PCR assay to determine SIV viral load was performed as previously described [38].

### SIV vRNA *in situ* hybridization, immunohistochemical, confocal microscopy and quantitative image analysis of brain tissue

To detect viral RNA in the tissues, in situ hybridization for SIV was performed using riboprobes as described previously [39]. Briefly, 7 µm-thick formalin-fixed, paraffin-embedded tissue sections were de-paraffinazed through a graded series of xylenes and ethanol and rehydrated in water threated with diethylpyrocarbonate (DEPC; Sigma; Aldrich, St. Louis, MO) before antigen retrieval by boiling in a microwave for 20 minutes in citrate buffer pH 6. The tissue sections were blocked with hybridization buffer containing 50% formamide with denatured herring sperm DNA and yeast tRNA at 10 mg/ml each in a humidified chamber at 45°C for 1 hr. Hybridization was performed with SIV-digoxigenin-labeled anti-sense riboprobes (Lofstrand Labs Ltd; Gaithersburg, MD) that were applied to the tissue sections at 10 ng/slide in hybridization buffer and incubated overnight at 45°C. After hybridization, slides were washed sequentially with 2× SSC, 1× SSC, and 0.1× SSC. The slides were incubated in TBS (tris buffer saline, 10 mM, pH 7.4) and followed with blocking solution for 1 h. Alkaline phosphatase-conjugated sheep anti-digoxigenin antibody diluted at 1∶200 (Roche; Penzberg, Germany) was used to detect hybridized digoxigenin-labeled probes. Either Dako Liquid Permanent Red or NBT/BICP substrate-chromogens system (Dako, Inc. Carpinteria, CA) were prepared according to manufacturer's instructions and added to the tissue for 20 min at room temp to develop the reaction. Controls included matched positive and negative tissues hybridized with digoxigenin-labeled sense RNA-labeled probes and processed in parallel.

Formalin-fixed, paraffin-embedded tissue sections (5–6 µm thick) were examined by immunohistochemical staining using the following primary antibodies: Anti CD163 (clone EDHu-1, AbD Serotec), HLA-DR (clone LN3, eBioscience), and PCNA (clone PC10, DAKO). Reactivity to primary antibodies was detected using the MACH3 alkaline phosphatase polymer detection kit from Biocare Medical (Concord, CA) with either NBT/BCIP substrate system for light microscopy or Permanent red for fluorescent microscopy both from Dako (Carpinteria, CA) after which sections were counterstained using YO-PRO nuclear stain (Life Technologies, Grand Island, NY). As controls, duplicate sections were processed in the absence of primary antibodies.

Triple label confocal microscopy was performed to co-localize SIV-RNA with cell type specific markers to determine the immunophenotype of infected cells as described previously [39]. Immunofluorescent labeling for microglia (rabbit polyclonal against IBA-1, Wako) and macrophages (Mouse IgG1 monoclonal to CD163, novocastra) was performed after ISH as previously described [15]. After incubation with the primary antibodies and subsequent washes, the appropriate species-specific secondary antibodies were applied; AlexaFluor 488 (green) conjugated goat-anti-rabbit (Invitrogen, Carlsbad, California) and AlexaFluor 647 (far-red, shown in blue) conjugated goat-anti-mouse IgG1 (Invitrogen, Carlsbad, California), respectively. To image the sections a Leica TCS SP2 confocal microscope equipped with 3 lasers (Leica Microsystems, Exton, PA) with a resolution of 512×512 pixels was used. The confocal imaging was performed using sequential mode to separately capture the fluorescence from the different fluorochromes. Volocity Software (v 5.5, Perkin-Elmer) was used to render the images from the Leica z stacks. Adobe Photoshop software (Version CS6; Adobe Systems) was used to assemble the images.

Quantitation of positive cells was performed manually for the SIV-RNA detection with NBT/BICP (purple-black color) by counting positive cells per field in 10 randomly selected fields encompassing a minimum of 120 mm^2^. The numbers of infected cells are expressed as cells per mm^2^. Quantitation of cells labeled for the cell type specific antigens was performed on immunolabeled slides using Inform v2 1.2 software after capturing 10 randomly selected fields encompassing a minimum of 30 mm^2^ with a CRI-multispectral camera using Nuance software v 3.0.2 (Perking-Elmer). The positive cell numbers are express as cells in mm^2^. Prism v5 software (GraphPad software) was used to present the data in a graphic form.

### SIV vRNA *in situ* hybridization, immunohistochemical analysis and quantitative image analysis in lymph node and intestinal tissues

Fluorescent in situ hybridization (F-ISH) was performed using our newly designed SIV riboprobes as previously published [7]. In brief, 5-µm tissue sections were mounted on Superfrost Plus Microscope Slides (Fisher Scientific), heated at 60°C for 1 h, dewaxed in xylenes and graded ethanols, and rehydrated in double-distilled H_2_O. Heat-induced epitope retrieval was performed by placing slides in in 10 mM Citrate (pH 6.0) containing 0.05% Tween-20 or Pretreatment-2 buffer (Advanced Cell Diagnostics, Inc.) at 100°C for 5 min followed by immediate immersion into HyPure molecular biology–grade H_2_O (Hyclone; Thermo Scientific) at room temperature. The slides were then incubated for 5 minutes at 37°C in a Tris-buffered solution containing 2 mM CaCI_2_ and proteinase K (1.25 µg/ml) then washed in HyPure molecular biology–grade H_2_O (Hyclone; Thermo Scientific), acetylated (0.25% acetic anhydride) for 20 minutes, and placed in 0.1 M triethanolamine (pH 8.0) until hybridization. Sections were then covered with hybridization solution (50% deionized formamide, 10% dextran sulfate, 0.6 M NaCI, 0.4 mg/ml yeast RNA (Ambion Inc.), and 1× Denhardt medium in 20 mM HEPES buffer (pH 7.2) with 1 mM EDTA) containing 100–400 ng/ml pooled SIV riboprobes and hybridized for 18 hours at 48°C. After hybridization, slides were washed in 5× SSC (1× SSC = 0.15 M NaCL+0.015 M Sodium Citrate) at 42°C for 20 minutes, 2× SSC in 50% formamide at 50°C for 20 minutes, and 1× RNA wash buffer (RWB: 0.1M TRIS-HCL PH 7.5, 0.4M NaCL, 0.05M EDTA PH 8.6) at 37°C with ribonuclease A (25 µg/ml) and T1 (25 U/ml) for 30 minutes. After washing in RWB buffer, 2× SSC, and 0.1× SSC at 37°C for 15 minutes each, sections were transferred to 1× Tris-buffered saline (TBS; Boston BioProducts) containing 0.05% Tween-20 (TBS-Tw). Tissues were blocked in TBS-Tw containing 2% donkey serum for 1 hour at room temperature, then incubated with goat anti-digoxigenin-DyLight-594 (Vector Labs; 1∶1,1000), mouse anti-CD68 (1∶400; clone KP1, Dako), mouse anti-CD163 (1∶400; clone 10D6; Novocastra/Leica), and rabbit monoclonal anti-CD3 (clone SP7; Labvision; 1∶200) in TBS-Tw containing 2% donkey serum overnight at 4°C. Slides were washed in TBS-Tw, incubated with donkey anti-goat–Alexa594, donkey anti-mouse–Alexa488, and donkey anti-rabbit–Alexa647 (all Invitrogen; 1∶400) for 1 hour at room temperature in the dark and washed in TBS-Tw. Slides were incubated with 0.1% Sudan Black B in 70% ethanol (Cat. No. 4410; ENG Scientific) and 1× TBS for 30 minutes at room temperature to quench autofluorescence then incubated with 300 nM DAPI for 10 min. Slides were washed, mounted with Prolong® Gold (Invitrogen) and imaged on an Olympus FV10i confocal microscope using a 60× phase contrast oil-immersion objective (NA 1.35) imaging using sequential mode to separately capture the fluorescence from the different fluorochromes at an image resolution of 1024×1024 pixels.

### Measurement of relative SIV vRNA abundance within distinct cell populations

Volumetric confocal images (Z-stack) were taken from lymph node and jejumal tissues following SIV F-ISH using the Nyquist sampling method using an Olympus FV10i confocal microscope with a 60× phase contrast oil-immersion objective (NA 1.35) with imaging using sequential mode to separately capture the fluorescence from the different fluorochromes at an image resolution of 1024×1024 pixels. Laser settings (gains and power) were set to ensure no pixel saturation in the SIV vRNA (Alexa594) channel and kept constant for all sample collections for each animal imaged. Images were opened in the Olympus FV10-ASW software (v3.1) and well-defined cellular regions were manually drawn around SIV vRNA^+^ macrophages and T cells from the compressed Z-stack image. The integrated intensity within these defined cell regions was measured in each Z-plane. The integrated intensity (or intensity integration) is the sum of the pixel intensities for a channel of interest within a specified object, and thus, the sum of the integrated intensities from each Z-stack is the total of the pixel intensities within each object volume. Thus, the “relative abundance” of SIV vRNA within the volume of each cell of interest was determined by summing the integrated intensities from each Z-plane.

### Plasma levels of sCD163

Soluble CD163 (sCD163) plasma levels were quantified by ELISA according to manufacturer's protocol (Trillium Diagnostics) using a 1∶50 dilution of plasma samples.

### Statistical analysis

Repeated-measures analyses for each outcome (CD4^+^ T cells; CD4^+^Ki-67^+^ T cells; CD14^+^CD16^+^; CD14^+^CD16^+^Ki-67^+^; and viral load) were performed with a means model with SAS Proc Mixed (version 9) providing separate estimates of the means by weeks post-depletion and infection between groups. A compound-symmetric variance-covariance form in repeated measurements was assumed for each outcome and robust estimates of the standard errors of parameters were used to perform statistical tests and construct 95% confidence intervals [40]. T-test or Mann Whitney test were used to compare the differences between the model-based treatment means (least-squares means) at each time point within the framework of the mixed effects linear model. Statistical tests were 2-sided. Pearson product-moment correlation coefficients were utilized to estimate linear associations for normally distributed data and Spearman rank correlation coefficients were used for skewed and other non-normal distributions. A P value ≤0.05 was considered statistically significant for the correlation analyses. The mean ± SEM were used as descriptive statistics for each continuous outcome. The rate of decay of virus was estimated by taking the linear slope of the natural log-transformed data of viral load from day 0 to 7 of treatment. The half-life is calculated as ln(2)/decay rate.

## Supporting Information

Figure S1 **Absolute levels of proliferating and CCR5^+^ CD4^+^ T cells are significantly lower in CD4-depleted SIV-infected RMs than in controls.** The absolute numbers of circulating CD4^+^ (**a**), CD4^+^Ki-67^+^ (**b**) or CD4^+^CCR5^+^ (**c**) T cells in severely depleted (orange square; n = 3), intermediately depleted (blue diamond; n = 5), and control (black circle; n = 4) RMs are shown. Total, proliferating, and CCR5^+^ T cell levels were similar in intermediately depleted and severely depleted animals, and significantly lower than those found in controls at all experimental time points (as assessed by repeated-measures analyses).
(TIFF)

Figure S2 **The level of soluble CD163 (sCD163) correlates with viremia in SIV-infected RMs.** Shown is the correlation between plasma levels of sCD163 and viremia (expressed as SIVmac_251_Gag RNA copies/mL) in all SIV-infected RMs (n = 12) included in the study. Statistical analyses were determined by Spearman rank correlation tests.
(TIFF)

Figure S3 **The number of TUNEL – but not active caspase 3 – positive cells in the brain is significantly higher in CD4-depleted SIV-infected RMs than in controls.** The number of cells staining positively for TUNEL (**a**) and active Caspase 3 (**b**) within brain tissue is shown for SIV-infected controls (closed circle; n = 3), CD4-depleted animals (orange square; n = 4), and CD4-depleted, ART-treated RMs (open square; n = 7).
(TIFF)

Table S1 **Survival of CD4 depleted SIV-infected RMs.** Antibody-mediated depletion of CD4 T cells prior to SIV infection results in fast disease progression, with seven out of eight RMs that required to be euthanized few days after initiation of ART. The table lists the day post-infection and post-ART initiation, as well as the CD4 count, at which each animal was sacrificed. *RVl11 survived throughout the entire study and was euthanized at day 234 post-infection. This animal completed 105 days of ART and was sacrificed at day 70 post ART-interruption.
(DOC)

## References

[1] Picker LJ, Maino VC (2000) The CD4(+) T cell response to HIV-1. Current opinion in immunology 12: 381–386. 10899031 10.1016/s0952-7915(00)00104-7

[2] Norris PJ, Rosenberg ES (2002) CD4(+) T helper cells and the role they play in viral control. Journal of molecular medicine 80: 397–405. 12110945 10.1007/s00109-002-0337-3

[3] Lackner AA, Lederman MM, Rodriguez B (2012) HIV pathogenesis: the host. Cold Spring Harbor perspectives in medicine 2: a007005. 22951442 10.1101/cshperspect.a007005 PMC3426821

[4] Klatt NR, Silvestri G (2012) CD4+ T cells and HIV: A paradoxical Pas de Deux. Science translational medicine 4: 123ps124. 10.1126/scitranslmed.3003862 22378922

[5] Haase AT (2010) Targeting early infection to prevent HIV-1 mucosal transmission. Nature 464: 217–223. 20220840 10.1038/nature08757

[6] Kelleher AD, Zaunders JJ (2006) Decimated or missing in action: CD4+ T cells as targets and effectors in the pathogenesis of primary HIV infection. Current HIV/AIDS reports 3: 5–12. 16522253 10.1007/s11904-006-0002-5

[7] Ortiz AM, Klatt NR, Li B, Yi Y, Tabb B, et al (2011) Depletion of CD4(+) T cells abrogates post-peak decline of viremia in SIV-infected rhesus macaques. The Journal of clinical investigation 121: 4433–4445. 22005304 10.1172/JCI46023 PMC3204830

[8] Francella N, Gwyn SE, Yi Y, Li B, Xiao P, et al (2013) CD4+ T cells support production of simian immunodeficiency virus env antibodies that enforce CD4-dependent entry and shape tropism in vivo. Journal of virology 87: 9719–9732. 23824793 10.1128/JVI.01254-13 PMC3754144

[9] Burdo TH, Soulas C, Orzechowski K, Button J, Krishnan A, et al (2010) Increased monocyte turnover from bone marrow correlates with severity of SIV encephalitis and CD163 levels in plasma. PLoS pathogens 6: e1000842. 20419144 10.1371/journal.ppat.1000842 PMC2855320

[10] Hasegawa A, Liu H, Ling B, Borda JT, Alvarez X, et al (2009) The level of monocyte turnover predicts disease progression in the macaque model of AIDS. Blood 114: 2917–2925. 19383966 10.1182/blood-2009-02-204263 PMC2756202

[11] Brown CR, Czapiga M, Kabat J, Dang Q, Ourmanov I, et al (2007) Unique pathology in simian immunodeficiency virus-infected rapid progressor macaques is consistent with a pathogenesis distinct from that of classical AIDS. Journal of virology 81: 5594–5606. 17376901 10.1128/JVI.00202-07 PMC1900277

[12] Burdo TH, Lentz MR, Autissier P, Krishnan A, Halpern E, et al (2011) Soluble CD163 made by monocyte/macrophages is a novel marker of HIV activity in early and chronic infection prior to and after anti-retroviral therapy. The Journal of infectious diseases 204: 154–163. 21628670 10.1093/infdis/jir214 PMC3105035

[13] Burdo TH, Weiffenbach A, Woods SP, Letendre S, Ellis RJ, et al (2013) Elevated sCD163 in plasma but not cerebrospinal fluid is a marker of neurocognitive impairment in HIV infection. Aids 27: 1387–1395. 23435298 10.1097/QAD.0b013e32836010bd PMC3844286

[14] Williams K, Schwartz A, Corey S, Orandle M, Kennedy W, et al (2002) Proliferating cellular nuclear antigen expression as a marker of perivascular macrophages in simian immunodeficiency virus encephalitis. The American journal of pathology 161: 575–585. 12163382 10.1016/S0002-9440(10)64213-7 PMC1850726

[15] Borda JT, Alvarez X, Mohan M, Hasegawa A, Bernardino A, et al (2008) CD163, a marker of perivascular macrophages, is up-regulated by microglia in simian immunodeficiency virus encephalitis after haptoglobin-hemoglobin complex stimulation and is suggestive of breakdown of the blood-brain barrier. The American journal of pathology 172: 725–737. 18276779 10.2353/ajpath.2008.070848 PMC2258269

[16] Perelson AS, Essunger P, Cao Y, Vesanen M, Hurley A, et al (1997) Decay characteristics of HIV-1-infected compartments during combination therapy. Nature 387: 188–191. 9144290 10.1038/387188a0

[17] Ho DD, Neumann AU, Perelson AS, Chen W, Leonard JM, et al (1995) Rapid turnover of plasma virions and CD4 lymphocytes in HIV-1 infection. Nature 373: 123–126. 7816094 10.1038/373123a0

[18] Wei X, Ghosh SK, Taylor ME, Johnson VA, Emini EA, et al (1995) Viral dynamics in human immunodeficiency virus type 1 infection. Nature 373: 117–122. 7529365 10.1038/373117a0

[19] Klatt NR, Shudo E, Ortiz AM, Engram JC, Paiardini M, et al (2010) CD8+ lymphocytes control viral replication in SIVmac239-infected rhesus macaques without decreasing the lifespan of productively infected cells. PLoS pathogens 6: e1000747. 20126441 10.1371/journal.ppat.1000747 PMC2813271

[20] Pierson T, McArthur J, Siliciano RF (2000) Reservoirs for HIV-1: mechanisms for viral persistence in the presence of antiviral immune responses and antiretroviral therapy. Annual review of immunology 18: 665–708. 10.1146/annurev.immunol.18.1.665 10837072

[21] Alexaki A, Liu YJ, Wigdahl B (2008) Cellular Reservoirs of HIV-1 and their Role in Viral Persistence. Curr Hiv Res 6: 388–400. 18855649 10.2174/157016208785861195 PMC2683678

[22] Igarashi T, Brown CR, Endo Y, Buckler-White A, Plishka R, et al (2001) Macrophage are the principal reservoir and sustain high virus loads in rhesus macaques after the depletion of CD4+ T cells by a highly pathogenic simian immunodeficiency virus/HIV type 1 chimera (SHIV): Implications for HIV-1 infections of humans. Proceedings of the National Academy of Sciences of the United States of America 98: 658–663. 11136236 10.1073/pnas.021551798 PMC14644

[23] Moller HJ (2012) Soluble CD163. Scandinavian journal of clinical and laboratory investigation 72: 1–13. 22060747 10.3109/00365513.2011.626868

[24] Burdo TH, Lackner A, Williams KC (2013) Monocyte/macrophages and their role in HIV neuropathogenesis. Immunological reviews 254: 102–113. 23772617 10.1111/imr.12068 PMC3704190

[25] Pulliam L, Gascon R, Stubblebine M, McGuire D, McGrath MS (1997) Unique monocyte subset in patients with AIDS dementia. Lancet 349: 692–695. 9078201 10.1016/S0140-6736(96)10178-1

[26] Fischer-Smith T, Croul S, Sverstiuk AE, Capini C, L'Heureux D, et al (2001) CNS invasion by CD14+/CD16+ peripheral blood-derived monocytes in HIV dementia: perivascular accumulation and reservoir of HIV infection. Journal of neurovirology 7: 528–541. 11704885 10.1080/135502801753248114

[27] Fischer-Smith T, Bell C, Croul S, Lewis M, Rappaport J (2008) Monocyte/macrophage trafficking in acquired immunodeficiency syndrome encephalitis: lessons from human and nonhuman primate studies. Journal of neurovirology 14: 318–326. 18780233 10.1080/13550280802132857 PMC2728912

[28] Clements JE, Mankowski JL, Gama L, Zink MC (2008) The accelerated simian immunodeficiency virus macaque model of human immunodeficiency virus-associated neurological disease: from mechanism to treatment. Journal of neurovirology 14: 309–317. 18780232 10.1080/13550280802132832 PMC8797541

[29] van Furth R (1989) Origin and turnover of monocytes and macrophages. Current topics in pathology Ergebnisse der Pathologie 79: 125–150. 2644082

[30] Kuroda MJ (2010) Macrophages: do they impact AIDS progression more than CD4 T cells? Journal of leukocyte biology 87: 569–573. 20053708 10.1189/jlb.0909626

[31] Brenchley JM, Paiardini M, Knox KS, Asher AI, Cervasi B, et al (2008) Differential Th17 CD4 T-cell depletion in pathogenic and nonpathogenic lentiviral infections. Blood 112: 2826–2835. 18664624 10.1182/blood-2008-05-159301 PMC2556618

[32] Sumpter B, Dunham R, Gordon S, Engram J, Hennessy M, et al (2007) Correlates of preserved CD4(+) T cell homeostasis during natural, nonpathogenic simian immunodeficiency virus infection of sooty mangabeys: implications for AIDS pathogenesis. Journal of immunology 178: 1680–1691. 10.4049/jimmunol.178.3.1680 17237418

[33] Engram JC, Cervasi B, Borghans JA, Klatt NR, Gordon SN, et al (2010) Lineage-specific T-cell reconstitution following in vivo CD4+ and CD8+ lymphocyte depletion in nonhuman primates. Blood 116: 748–758. 20484087 10.1182/blood-2010-01-263814 PMC2918331

[34] Paiardini M, Cervasi B, Engram JC, Gordon SN, Klatt NR, et al (2009) Bone marrow-based homeostatic proliferation of mature T cells in nonhuman primates: implications for AIDS pathogenesis. Blood 113: 612–621. 18832134 10.1182/blood-2008-06-159442 PMC2628369

[35] Paiardini M, Cervasi B, Reyes-Aviles E, Micci L, Ortiz AM, et al (2011) Low levels of SIV infection in sooty mangabey central memory CD4(+) T cells are associated with limited CCR5 expression. Nat Med 17: 830-U197. 21706028 10.1038/nm.2395 PMC3253129

[36] Micci L, Cervasi B, Ende ZS, Iriele RI, Reyes-Aviles E, et al (2012) Paucity of IL-21-producing CD4(+) T cells is associated with Th17 cell depletion in SIV infection of rhesus macaques. Blood 120: 3925–3935. 22990011 10.1182/blood-2012-04-420240 PMC3496953

[37] Pallikkuth S, Micci L, Ende ZS, Iriele RI, Cervasi B, et al (2013) Maintenance of intestinal Th17 cells and reduced microbial translocation in SIV-infected rhesus macaques treated with interleukin (IL)-21. PLoS pathogens 9: e1003471. 23853592 10.1371/journal.ppat.1003471 PMC3701718

[38] Amara RR, Villinger F, Altman JD, Lydy SL, O'Neil SP, et al (2001) Control of a mucosal challenge and prevention of AIDS by a multiprotein DNA/MVA vaccine. Science 292: 69–74. 11393868 10.1126/science.1058915

[39] Borda JT, Alvarez X, Kondova I, Aye P, Simon MA, et al (2004) Cell tropism of simian immunodeficiency virus in culture is not predictive of in vivo tropism or pathogenesis. American Journal of Pathology 165: 2111–2122. 15579453 10.1016/S0002-9440(10)63261-0 PMC1618703

[40] Diggle PJ, Liang KY and Zeger SL (1994). Analysis of longitudinal data. Oxford UK: Clarendon Press.

