# Supplementary figures and images for: CD4 Depletion in SIV-Infected Macaques Results in Macrophage and Microglia Infection with Rapid Turnover of Infected Cells
Source: PLoS Pathog. 2014 Oct 30;10(10):e1004467. doi: 10.1371/journal.ppat.1004467 (PMC4214815; doi:10.1371/journal.ppat.1004467)

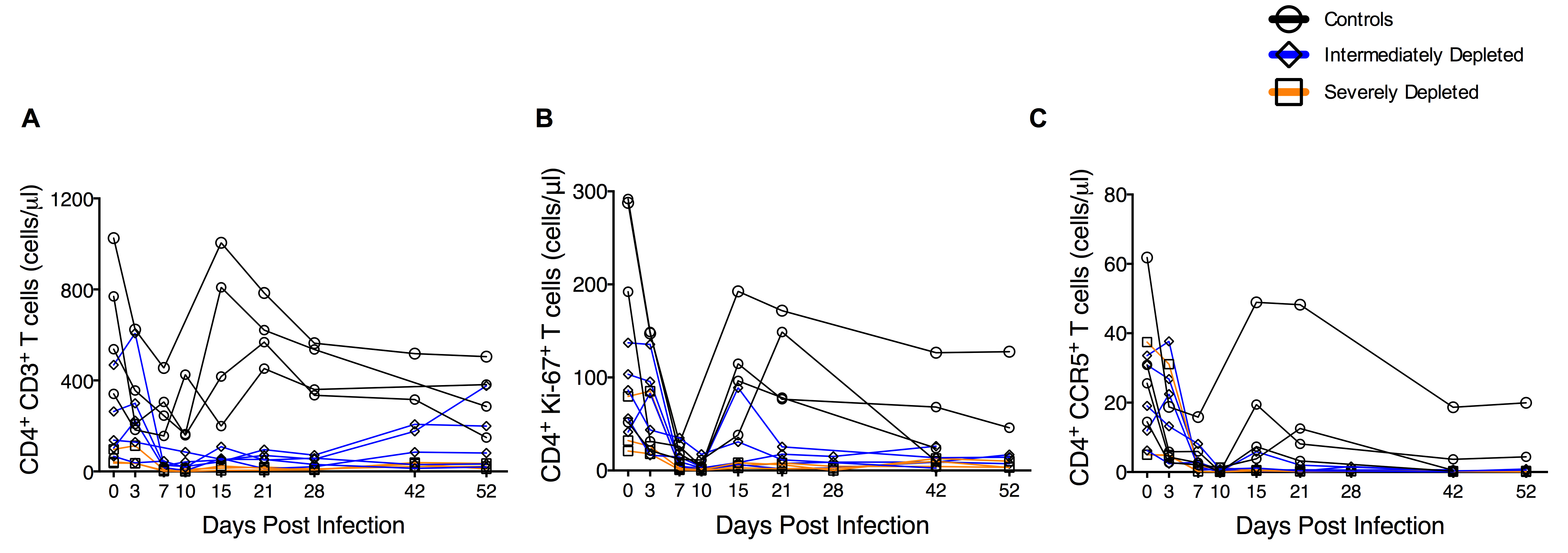

Supplement: Figure S1 — Absolute levels of proliferating and CCR5+ CD4+ T cells are significantly lower in CD4-depleted SIV-infected RMs than in controls. The absolute numbers of circulating CD4+ (a), CD4+Ki-67+ (b) or CD4+CCR5+ (c) T cells in severely depleted (orange square; n = 3), intermediately depleted (blue diamond; n = 5), and control (black circle; n = 4) RMs are shown. Total, proliferating, and CCR5+ T cell levels were similar in intermediately depleted and severely depleted animals, and significantly lower than those found in controls at all experimental time points (as assessed by repeated-measures analyses). (TIFF) [file ppat.1004467.s001.tiff]

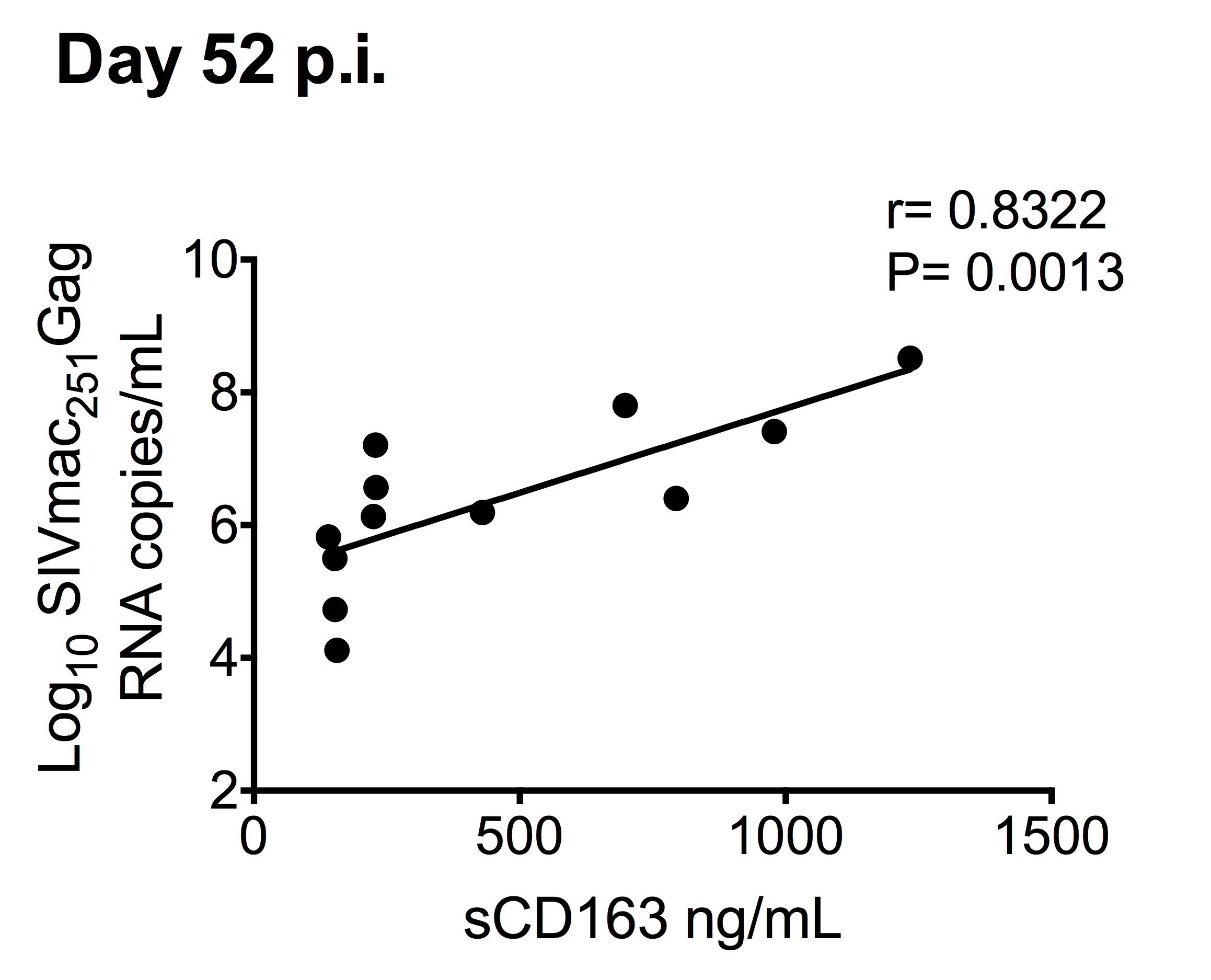

Supplement: Figure S2 — The level of soluble CD163 (sCD163) correlates with viremia in SIV-infected RMs. Shown is the correlation between plasma levels of sCD163 and viremia (expressed as SIVmac251Gag RNA copies/mL) in all SIV-infected RMs (n = 12) included in the study. Statistical analyses were determined by Spearman rank correlation tests. (TIFF) [file ppat.1004467.s002.tiff]

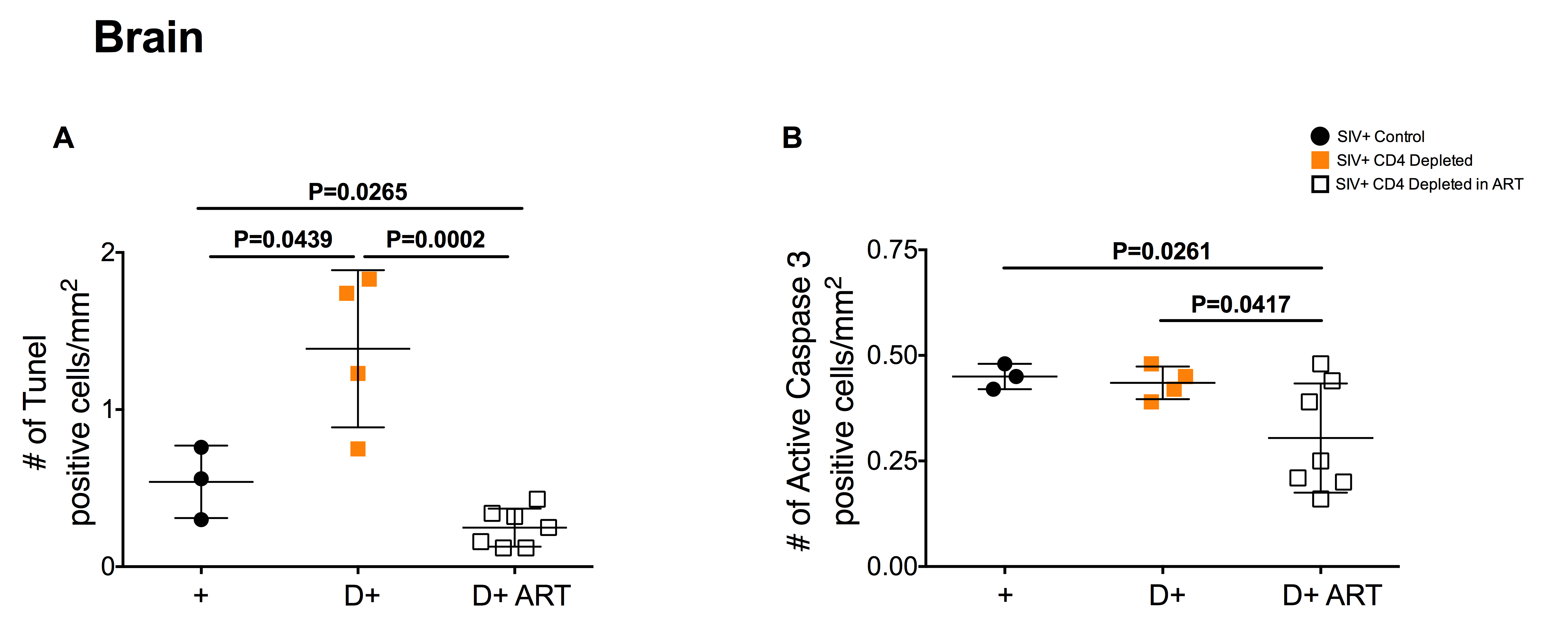

Supplement: Figure S3 — The number of TUNEL – but not active caspase 3 – positive cells in the brain is significantly higher in CD4-depleted SIV-infected RMs than in controls. The number of cells staining positively for TUNEL (a) and active Caspase 3 (b) within brain tissue is shown for SIV-infected controls (closed circle; n = 3), CD4-depleted animals (orange square; n = 4), and CD4-depleted, ART-treated RMs (open square; n = 7). (TIFF) [file ppat.1004467.s003.tiff]
