# Supplementary material for: CD4 Depletion in SIV-Infected Macaques Results in Macrophage and Microglia Infection with Rapid Turnover of Infected Cells
Source: PLoS Pathog. 2014 Oct 30;10(10):e1004467. doi: 10.1371/journal.ppat.1004467 (PMC4214815; doi:10.1371/journal.ppat.1004467)
Supplement: Table S1 — Survival of CD4 depleted SIV-infected RMs. Antibody-mediated depletion of CD4 T cells prior to SIV infection results in fast disease progression, with seven out of eight RMs that required to be euthanized few days after initiation of ART. The table lists the day post-infection and post-ART initiation, as well as the CD4 count, at which each animal was sacrificed. *RVl11 survived throughout the entire study and was euthanized at day 234 post-infection. This animal completed 105 days of ART and was sacrificed at day 70 post ART-interruption. (DOC) [file ppat.1004467.s004.doc]

**Supplementary table 1: Survival of CD4 depleted SIV-infected rhesus macaques**

**at necropsy**

| **Animal code** | **CD4 depletion status** | **Day post-infection**  **at necropsy** | **Day on-ART**  **at necropsy** |
| --- | --- | --- | --- |
| RVo10 | Intermediately | 55 | 3 |
| REc7 | Severely | 58 | 6 |
| RYk10 | Severely | 62 | 10 |
| RTi7 | Intermediately | 64 | 12 |
| RQk8 | Severely | 64 | 12 |
| RQb8 | Intermediately | 81 | 30 |
| RMv9 | Intermediately | 99 | 48 |
| RVl11***** | Intermediately | 234 | N.A. |
|  |  |  |  |
|  |  |  |  |
|  |  |  |  |

**CD4 count**

176

41

44

168

77

176

83

410
